# Crystal structure, Hirshfeld surface analysis, inter­action energy and DFT studies of 4-[(4-allyl-2-meth­oxy­phen­oxy)meth­yl]-1-(4-meth­oxy­phen­yl)-1*H*-1,2,3-triazole

**DOI:** 10.1107/S2056989020006994

**Published:** 2020-05-29

**Authors:** Abdelmaoujoud Taia, Mohamed Essaber, Abdeljalil Aatif, Karim Chkirate, Tuncer Hökelek, Joel T. Mague, Nada Kheira Sebbar

**Affiliations:** a Laboratory of Molecular Chemistry, Department of Chemistry, Faculty of Sciences Semlalia, University of Cadi Ayyad, BP 2390, 40001 Marrakech, Morocco; b Laboratoire de Chimie Organique Heterocyclique URAC 21, Pôle de Competence Pharmacochimie, Av. Ibn Battouta, BP 1014, Faculté des Sciences, Université Mohammed V, Rabat, Morocco; c Department of Physics, Hacettepe University, 06800 Beytepe, Ankara, Turkey; d Department of Chemistry, Tulane University, New Orleans, LA 70118, USA; e Laboratoire de Chimie Appliquée et Environnement, Equipe de Chimie Bioorganique Appliquée, Faculté des Sciences, Université Ibn Zohr, Agadir, Morocco

**Keywords:** crystal structure, triazole, hydrogen bonding, C—H⋯π(ring) inter­action, π-stacking

## Abstract

In the title mol­ecule, C_20_H_21_N_3_O_3_, the allyl substituent is rotated out of the plane of its attached phenyl ring [torsion angle 100.66 (15)°]. In the crystal, C—H_Mthphn_⋯O_Mthphn_ (Mthphn = meth­oxy­phen­yl) hydrogen bonds lead to the formation of (100) layers that are connected into a three-dimensional network by C—H⋯π(ring) inter­actions, together with π–π stacking inter­actions [centroid-to-centroid distance = 3.7318 (10) Å] between parallel phenyl rings. Hirshfeld surface analysis indicates that the most important contributions to the crystal packing are from H⋯H (48.7%) and H⋯C/C⋯H (23.3%) inter­actions. Computational chemistry reveals that the C—H_Mthphn_⋯O_Mthphn_ hydrogen bond energy is 47.1 kJ mol^−1^. The theoretical structure, optimized by density functional theory (DFT) at the B3LYP/ 6–311 G(d,p) level, is compared with the experimentally determined mol­ecular structure. The HOMO–LUMO behaviour was elucidated to determine the energy gap.

## Abstract 2

In the crystal structure, C—H_Mthphn_⋯O_Mthphn_ (Mthphn = meth­oxy­phen­yl) hydrogen bonds form corrugated layers parallel to (100) that are connected along the *a* axis by C—H⋯π(ring) and π–π stacking inter­actions.

## Chemical context   

Clove essential oil is extracted from cloves, which come from a tree belonging to the Myrtaceae family (Chang & Miau, 1984 ▸), originating from the Moluccas in Indonesia. Eugenol (C_10_H_12_O_2_) is the major constituent of clove essential oil with a percentage of 75–90% (Patra & Saxena, 2010 ▸). Eugenol is a mol­ecule that belongs to the family of phenyl­propenes; its aromatic ring, an alcohol function and an allylic entity explain its high reactivity. Several studies have revealed various biological activities for eugenol, including anti­viral (Benencia & Courreges, 2000 ▸), anti-leishmania (Ueda-Nakamura *et al.*, 2006 ▸), anti­bacterial (Pathirana *et al.*, 2019 ▸), anti­fungal (Wang *et al.*, 2010 ▸), anti-inflammatory (Daniel *et al.*, 2009 ▸), anti­oxidant (Mahboub & Memmou., 2015 ▸), anesthetic analgesic (Guenette *et al.*, 2007 ▸), anti­cancer (Hussain *et al.*, 2011 ▸) or anti-diabetes (Mnafgui *et al.*, 2013 ▸) properties. On the other hand, 1,2,3-triazoles are known by their diverse biological activities being used as anti­leishmania (Teixeira *et al.*, 2018 ▸), anti­microbial (Glowacka *et al.*, 2019 ▸) or anti­viral (Bankowska, *et al.*, 2014 ▸) agents. In this context, we have synthesized the title compound, (I), through cyclo­addition reaction of 1-azido-4-meth­oxy­benzene with 4-allyl-2-meth­oxy-1-(prop-2-yn­yloxy) benzene; the latter was previously prepared by O-alkyl­ation of eugenol by propargile (Taia *et al.*, 2020 ▸).
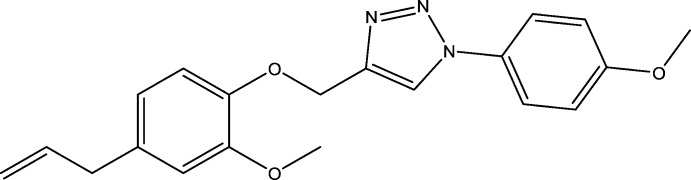


We report herein the synthesis, mol­ecular and crystal structures of (I), along with the results of a Hirshfeld surface analysis, an inter­action energy calculation, and a density functional theory (DFT) study.

## Structural commentary   

**Figure 1:**
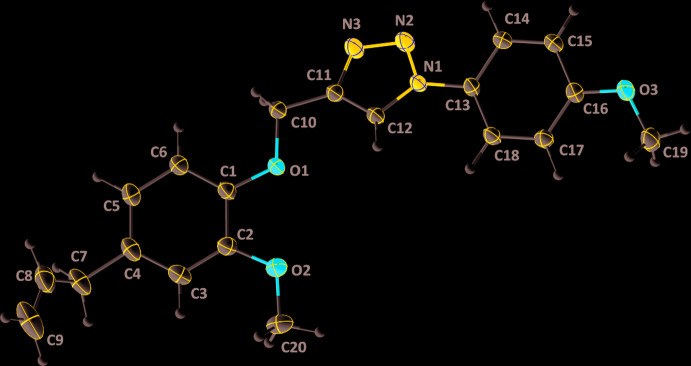
The mol­ecular structure of (I) with the atom-numbering scheme. Displacement ellipsoids are drawn at the 50% probability level.

The title mol­ecule is non-planar (Fig. 1 ▸), with the *A* (C1–C6) and *C* (C13–C18) benzene rings inclined to the *B* (C11/C12/N1–N3) triazole ring by 25.76 (4) and 24.97 (4)°, respectively. The allyl group is rotated out of the plane of the *A* ring as indicated by the C3—C4—C7—C8 torsion angle of 100.66 (15)°. Both meth­oxy groups are virtually coplanar with their attached rings with C3—C2—O2—C20 and C17—C16—O3—C19 torsion angles, respectively, of 5.04 (16) and 3.73 (16)°. There are no unusual bond lengths or bond angles in the mol­ecule.

## Supra­molecular features   

**Figure 2:**
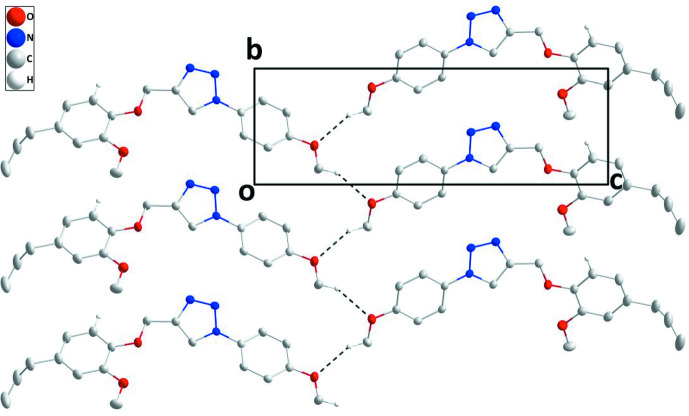
A portion of one layer viewed along the *a* axis, with C—H_Mthphn_⋯O_Mthphn_ (Mthphn = meth­oxy­phen­yl) hydrogen bonds depicted by dashed lines.

**Figure 3:**
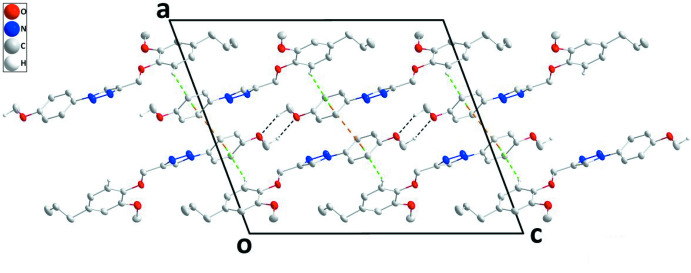
Projection of the crystal structure along the *b* axis. C—H_Mthphn_⋯O_Mthphn_ (Mthphn = meth­oxy­phen­yl) hydrogen bonds and π–π stacking and C—H⋯π(ring) inter­actions are depicted, respectively, by black, orange and green dashed lines.

In the crystal structure, (100) layers are formed by C—H_Mthphn_⋯O_Mthphn_ (Mthphn = meth­oxy­phen­yl) hydrogen bonds (Table 1 ▸, Fig. 2 ▸). These are stacked along the *a* axis through C6—H6⋯*Cg*3(*x*, −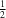 − *y*, −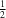 + *z*) inter­actions (Table 1 ▸) as well as through π—-π stacking inter­actions between inversion-related *C* rings [*Cg*3⋯*Cg*3(1 − *x*, −*y*, 1 − *z*] with a centroid-to-centroid distance of 3.7318 (10) Å (Fig. 3 ▸).

## Hirshfeld surface analysis   

**Figure 4:**
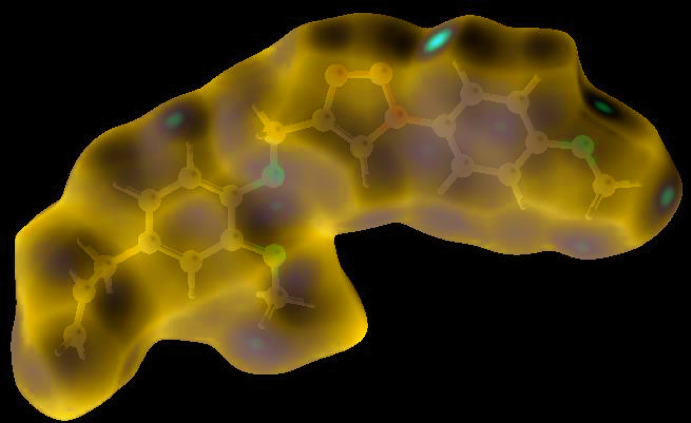
View of the three-dimensional Hirshfeld surface of the title compound plotted over *d*_norm_ in the range of −0.2587 to 1.3813 a.u..

**Figure 5:**
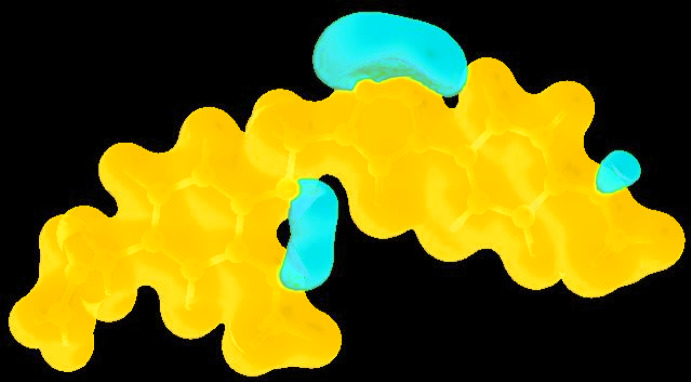
View of the three-dimensional Hirshfeld surface of the title compound plotted over electrostatic potential energy in the range −0.0500 to 0.0500 a.u..

**Figure 6:**
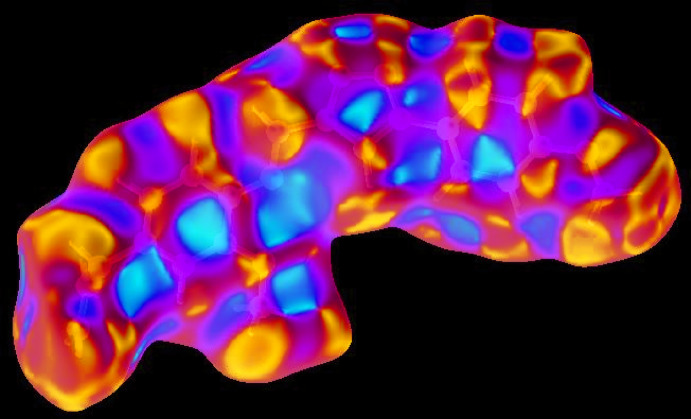
Hirshfeld surface of the title compound plotted over shape-index.

In order to visualize and qu­antify the inter­molecular inter­actions in the crystal of (I), a Hirshfeld surface (HS) analysis (Hirshfeld, 1977 ▸; Spackman & Jayatilaka, 2009 ▸) was carried out by using *Crystal Explorer 17.5* (Turner *et al.*, 2017 ▸). In the HS plotted over *d*_norm_ (Fig. 4 ▸), the white surface indicates contacts with distances equal to the sum of van der Waals radii, and the red and blue colours indicate distances shorter or longer than the van der Waals radii, respectively (Venkatesan *et al.*, 2016 ▸). The bright-red spots appearing near hydrogen atoms (H6 and H19*B*), and near O3 indicate their roles in hydrogen bonding; they also appear as blue and red regions corresponding to positive (hydrogen-bond donors) and negative (hydrogen-bond acceptors) potentials on the HS mapped over electrostatic potential (Spackman *et al.*, 2008 ▸; Jayatilaka *et al.*, 2005 ▸), as shown in Fig. 5 ▸. The HS plotted over the shape-index (Fig. 6 ▸) clearly reveals π–π stacking inter­actions (visualized as red and blue areas) in (I), as discussed above.

**Figure 7:**
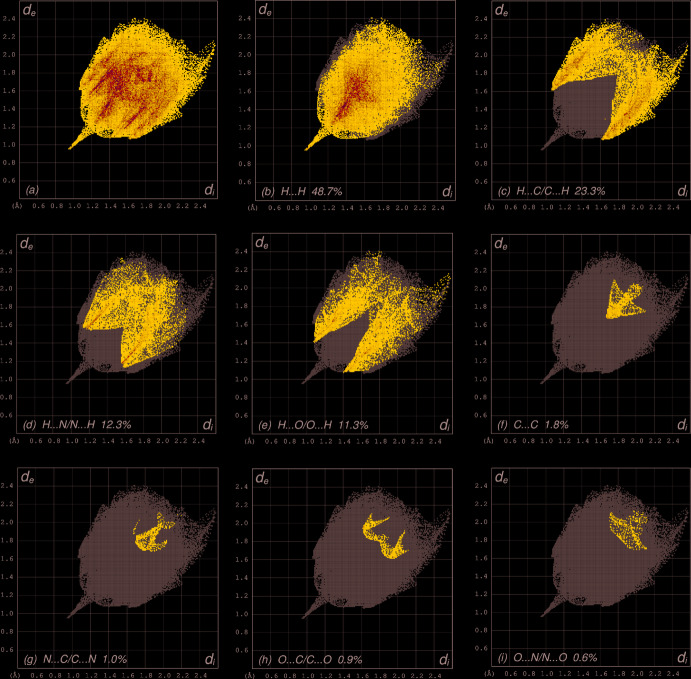
Two-dimensional fingerprint plots for (I), showing (*a*) all inter­actions, and delineated into (*b*) H⋯H, (*c*) H⋯C/C⋯H, (*d*) H⋯N/N⋯H, (*e*) H⋯O/O⋯H, (*f*) C⋯C, (*g*) N⋯C/C⋯N, (*h*) O⋯C/C⋯O and (i) O⋯N/N⋯O inter­actions. *d*_i_ and *d*_e_ refer to the closest inter­nal and external distances (in Å) from given points on the Hirshfeld surface contacts.

The overall two-dimensional fingerprint plot, Fig. 7 ▸ *a*, and those delineated into H⋯H, H⋯C/C⋯H, H⋯N/N⋯H, H⋯O/O⋯H, C⋯C, N⋯C/C⋯N, O⋯C/C⋯O and O⋯N/N⋯O contacts (McKinnon *et al.*, 2007 ▸) are illustrated in Fig. 7 ▸ *b*–*i*, respectively, together with their relative contributions to the Hirshfeld surface. The most important inter­action is H⋯H contributing 48.7% to the overall crystal packing, which is reflected in Fig. 7 ▸ *b* as widely scattered points of high density due to the large hydrogen content of the mol­ecule with the tip at *d*_e_ = *d*_i_ = 0.95 Å. In the presence of C—H⋯π inter­actions, the pair of characteristic wings of H⋯C/C⋯H contacts (23.3% contribution to the HS, Fig. 7 ▸ *c*) has the tips at *d*_e_ + *d*_i_ = 2.68 Å. The pair of scattered points of spikes in the fingerprint plot delineated into H⋯N/N⋯H contacts (12.3% contribution, Fig. 7 ▸ *d*) has a distribution of points with small and slightly larger tips at *d*_e_ + *d*_i_ = 2.72 and 2.70 Å, respectively. The H⋯O/O⋯H contacts (Fig. 7 ▸ *e*, 11.3% contribution) have a symmetric distribution of points with the tips at *d*_e_ + *d*_i_ = 2.48 Å. The C⋯C contacts, Fig. 7 ▸ *f*, have an arrow-shaped distribution of points with the tip at *d*_e_ = *d*_i_ = 1.68 Å. Finally, N⋯C/C⋯N (Fig. 7 ▸ *g*), O⋯C/C⋯O (Fig. 7 ▸ *h*) and O⋯N/N⋯O (Fig. 7 ▸ *i*) inter­actions contribute only 1.0%, 0.9% and 0.6%, respectively, to the overall HS and thus have minor significance.

The Hirshfeld surface analysis confirms the importance of H-atom contacts in establishing the packing. The large number of H⋯H and H⋯C/C⋯H inter­actions suggest that van der Waals inter­actions and hydrogen bonding play the major roles in the crystal packing (Hathwar *et al.*, 2015 ▸).

## Inter­action energy calculations   

The inter­molecular inter­action energies were calculated using a CE–B3LYP/6–31G(d,p) energy model available in *Crystal Explorer 17.5* (Turner *et al.*, 2017 ▸), where a cluster of mol­ecules was generated within a radius of 3.8 Å by default (Turner *et al.*, 2014 ▸). The total inter­molecular energy (*E*_tot_) is the sum of electrostatic (*E*_ele_), polarization (*E*_pol_), dispersion (*E*_dis_) and exchange-repulsion (*E*_rep_) energies (Turner *et al.*, 2015 ▸) with scale factors of 1.057, 0.740, 0.871 and 0.618, respectively (Mackenzie *et al.*, 2017 ▸). In (I), the relevant C19—H19*B*⋯O3 hydrogen-bonding inter­action energies (in kJ mol^−1^) were calculated as −20.6 (*E*_ele_), −5.7 (*E*_pol_), −49.3 (*E*_dis_), 35.4 (*E*_rep_) and −47.1 (*E*_tot_).

## DFT calculations   

Density functional theory (DFT) using standard B3LYP functional and 6–311 G(d,p) basis-set calculations (Becke, 1993 ▸) as implemented in *GAUSSIAN 09* (Frisch *et al.*, 2009 ▸) was used to optimize the mol­ecular structure of (I) in the gas phase. Theoretical and experimental results in terms of bond lengths and angles are in good agreement (Table 2 ▸).

**Figure 8:**
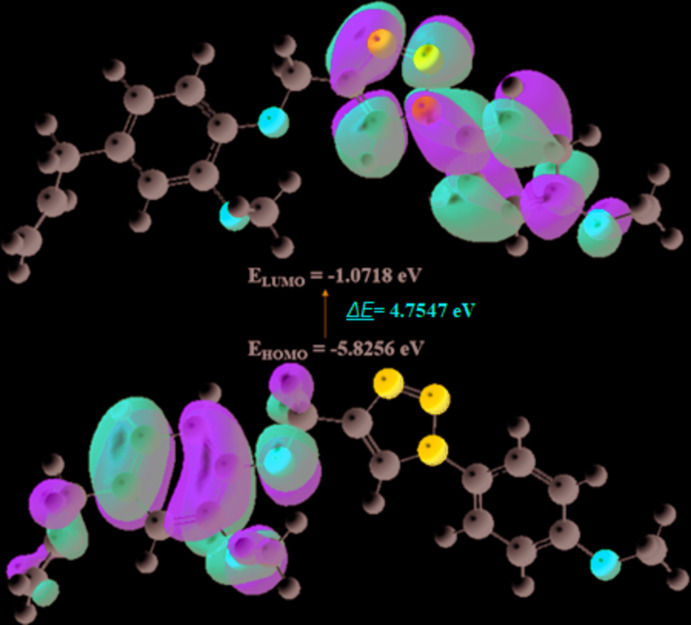
HOMO and LUMO of (I), and the energy band gap between them.

The highest-occupied mol­ecular orbital (HOMO) and the lowest-unoccupied mol­ecular orbital (LUMO) together with the energy gap between them (Δ*E* = *E*_LUMO_ – *E*_HOMO_) are shown in Fig. 8 ▸. Table 3 ▸ collates calculated energies, including those for *E*_HOMO_ and *E*_LUMO_, electronegativity (χ), hardness (η), potential (μ), electrophilicity (ω) and softness (*σ*).

## Database survey   

An eugenol 4-allyl-2-meth­oxy­phenol analogue has been reported by Ghosh *et al.* (2005 ▸). Others similar compounds have also been reported (Ogata *et al.*, 2000 ▸; Yoo *et al.*, 2005 ▸; Sadeghian *et al.*, 2008 ▸; Ma *et al.* 2010 ▸).

## Synthesis and crystallization   

To a solution of 4-allyl-2-meth­oxy-1-(prop-2-yn­yloxy) benzene (0.4 ml, 2.5 mmol) in anhydrous aceto­nitrile, 1-azido-4-meth­oxy­benzene (0.30 ml, 2.5 mmol) and 10 mg copper (I) iodide (CuI) were added. The mixture was refluxed for 2 h. After cooling, the reaction mixture was extracted three times with di­chloro­methane. The organic phase was dried with sodium sulfate and purified by column chromatography on silica gel, eluent hexa­ne–ethyl acetate (*v*/*v* = 80/20). Colourless crystals were isolated when the solvent was allowed to evaporate (yield: 88%).

## Refinement   

Crystal data, data collection and structure refinement details are summarized in Table 4 ▸. Hydrogen atoms were located in a difference-Fourier map and were refined freely.

## Supplementary Material

Crystal structure: contains datablock(s) I, global. DOI: 10.1107/S2056989020006994/wm5559sup1.cif

Structure factors: contains datablock(s) I. DOI: 10.1107/S2056989020006994/wm5559Isup2.hkl

Supporting information file. DOI: 10.1107/S2056989020006994/wm5559Isup3.cdx

CCDC reference: 2005277

Additional supporting information:  crystallographic information; 3D view; checkCIF report

## Figures and Tables

**Table 1:** Hydrogen-bond geometry (Å, °) *Cg*3 is the centroid of the benzene ring *C* (C13–C18).

*D*—H⋯*A*	*D*—H	H⋯*A*	*D*⋯*A*	*D*—H⋯*A*
C6—H6⋯*Cg*3^xiii^	0.964 (15)	2.825 (15)	3.5168 (15)	129.4 (11)
C19—H19*B*⋯O3^xiv^	0.977 (18)	2.578 (18)	3.4587 (16)	150.0 (14)

Symmetry codes: (xiii) 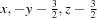; (xiv) 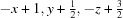.

**Table 2:** Comparison of selected bond length and angles (Å, °) between exerimental data (X-ray) and theory [B3LYP/6–311G(d,p)]

Bonds/angles	X-ray	B3LYP/6–311G(d,p)
O1—C1	1.3712 (12)	1.39510
O1—C10	1.4279 (12)	1.45830
O2—C2	1.3673 (13)	1.39818
O2—C20	1.4220 (14)	1.46747
O3—C16	1.3631 (12)	1.38746
O3—C19	1.4213 (15)	1.45298
N1—N2	1.3504 (13)	1.39727
N1—C12	1.3541 (13)	1.36977
N1—C13	1.4315 (13)	1.42427
N2—N3	1.3142 (13)	1.32619
N3—C11	1.3600 (13)	1.38002
C8—C9	1.312 (2)	1.33811
		
C1—O1—C10	117.19 (8)	117.72628
C2—O2—C20	117.19 (10)	117.20245
C16—O3—C19	117.42 (9)	118.93805
N2—N1—C12	110.77 (8)	110.09008
N2—N1—C13	119.89 (8)	120.52180
C12—N1—C13	129.33 (9)	129.38444
N3—N2—N1	107.21 (8)	106.61104
N2—N3—C11	108.86 (9)	109.15766
O1—C1—C6	125.33 (9)	124.33053

**Table 3:** Calculated energies and other parameters for (I)

Total Energy *TE* (eV)	−31679.5273
*E*_HOMO_ (eV)	−5.8256
*E*_LUMO_ (eV)	−1.0718
Gap, *ΔE* (eV)	4.7547
Dipole moment, *μ* (Debye)	2.6382
Ionization potential, *I* (eV)	5.8256
Electron affinity, *A*	1.0718
Electronegativity, *χ*	3.4491
Hardness, *η*	2.3773
Electrophilicity index, *ω*	2.5021
Softness, *σ*	0.4206
Fraction of electron transferred, *ΔN*	0.7468

**Table 4:** Experimental details

Crystal data	
Chemical formula	C_20_H_21_N_3_O_3_
*M*_r_	351.40
Crystal system, space group	Monoclinic, *P*2_1_/*c*
Temperature (K)	150
*a*, *b*, *c* (Å)	16.212 (3), 5.9584 (12), 19.450 (4)
β (°)	110.537 (3)
*V* (Å^3^)	1759.5 (6)
*Z*	4
Radiation type	Mo *K*α
μ (mm^−1^)	0.09
Crystal size (mm)	0.38 × 0.33 × 0.32

Data collection	
Diffractometer	Bruker *SMART* *APEX* CCD
Absorption correction	Multi-scan (*SADABS*; Krause *et al.*, 2015 ▸)
*T*_min_, *T*_max_	0.88, 0.97
No. of measured, independent and observed [*I* > 2σ(*I*)] reflections	32548, 4788, 3978
*R*_int_	0.027
(sin θ/λ)_max_ (Å^−1^)	0.689

Refinement	
*R*[*F* ^2^ > 2σ(*F* ^2^)], *wR*(*F* ^2^), *S*	0.044, 0.132, 1.09
No. of reflections	4788
No. of parameters	319
H-atom treatment	All H-atom parameters refined
Δρ_max_, Δρ_min_ (e Å^−3^)	0.54, −0.22

Computer programs: *APEX3* and *SAINT* (Bruker, 2016 ▸), *SHELXT* (Sheldrick, 2015*a* ▸), *SHELXL2018/1* (Sheldrick, 2015*b* ▸), *DIAMOND* (Brandenburg & Putz, 2012 ▸) and*publCIF* (Westrip, 2010 ▸).

## supplementary crystallographic information

### Crystal data

**Table:**

C_20_H_21_N_3_O_3_	*F*(000) = 744
*M**_r_* = 351.40	*D*_x_ = 1.327 Mg m^−^^3^
Monoclinic, *P*2_1_/*c*	Mo *K*α radiation, λ = 0.71073 Å
*a* = 16.212 (3) Å	Cell parameters from 9905 reflections
*b* = 5.9584 (12) Å	θ = 2.2–29.3°
*c* = 19.450 (4) Å	µ = 0.09 mm^−^^1^
β = 110.537 (3)°	*T* = 150 K
*V* = 1759.5 (6) Å^3^	Block, colourless
*Z* = 4	0.38 × 0.33 × 0.32 mm

### Data collection

**Table:**

Bruker SMART APEX CCD diffractometer	4788 independent reflections
Radiation source: fine-focus sealed tube	3978 reflections with *I* > 2σ(*I*)
Graphite monochromator	*R*_int_ = 0.027
Detector resolution: 8.3333 pixels mm^-1^	θ_max_ = 29.3°, θ_min_ = 2.2°
φ and ω scans	*h* = −22→21
Absorption correction: multi-scan (*SADABS*; Krause *et al.*, 2015)	*k* = −8→8
*T*_min_ = 0.88, *T*_max_ = 0.97	*l* = −26→26
32548 measured reflections	

### Refinement

**Table:**

Refinement on *F*^2^	Primary atom site location: dual
Least-squares matrix: full	Secondary atom site location: difference Fourier map
*R*[*F*^2^ > 2σ(*F*^2^)] = 0.044	Hydrogen site location: difference Fourier map
*wR*(*F*^2^) = 0.132	All H-atom parameters refined
*S* = 1.09	*w* = 1/[σ^2^(*F*_o_^2^) + (0.0858*P*)^2^ + 0.1761*P*] where *P* = (*F*_o_^2^ + 2*F*_c_^2^)/3
4788 reflections	(Δ/σ)_max_ < 0.001
319 parameters	Δρ_max_ = 0.54 e Å^−^^3^
0 restraints	Δρ_min_ = −0.22 e Å^−^^3^

### Special details

**Table:**

Experimental. The diffraction data were obtained from 3 sets of 400 frames, each of width 0.5° in ω, colllected at φ = 0.00, 90.00 and 180.00° and 2 sets of 800 frames, each of width 0.45° in φ, collected at ω = –30.00 and 210.00°. The scan time was 10 sec/frame.
Geometry. All esds (except the esd in the dihedral angle between two l.s. planes) are estimated using the full covariance matrix. The cell esds are taken into account individually in the estimation of esds in distances, angles and torsion angles; correlations between esds in cell parameters are only used when they are defined by crystal symmetry. An approximate (isotropic) treatment of cell esds is used for estimating esds involving l.s. planes.
Refinement. Refinement of F^2^ against ALL reflections. The weighted R-factor wR and goodness of fit S are based on F^2^, conventional R-factors R are based on F, with F set to zero for negative F^2^. The threshold expression of F^2^ > 2sigma(F^2^) is used only for calculating R-factors(gt) etc. and is not relevant to the choice of reflections for refinement. R-factors based on F^2^ are statistically about twice as large as those based on F, and R- factors based on ALL data will be even larger.

### Fractional atomic coordinates and isotropic or equivalent isotropic displacement parameters (Å^2^)

**Table:**

	*x*	*y*	*z*	*U*_iso_*/*U*_eq_	
O1	0.22952 (5)	−0.13314 (13)	0.17036 (4)	0.02658 (18)	
O2	0.13208 (6)	0.21761 (14)	0.12923 (5)	0.0343 (2)	
O3	0.45455 (5)	0.16414 (14)	0.66870 (4)	0.03039 (19)	
N1	0.35850 (6)	−0.22912 (14)	0.39234 (4)	0.02175 (18)	
N2	0.36971 (7)	−0.45263 (15)	0.38817 (5)	0.0295 (2)	
N3	0.34449 (6)	−0.50475 (15)	0.31817 (5)	0.0293 (2)	
C1	0.19702 (6)	−0.09553 (18)	0.09610 (5)	0.0237 (2)	
C2	0.14239 (7)	0.09424 (18)	0.07352 (6)	0.0253 (2)	
C3	0.10348 (7)	0.14141 (19)	−0.00049 (6)	0.0298 (2)	
H3	0.0643 (10)	0.270 (3)	−0.0155 (8)	0.039 (4)*	
C4	0.11724 (7)	0.0034 (2)	−0.05366 (6)	0.0314 (2)	
C5	0.17275 (8)	−0.1789 (2)	−0.03076 (6)	0.0331 (3)	
H5	0.1821 (11)	−0.278 (3)	−0.0671 (9)	0.043 (4)*	
C6	0.21292 (7)	−0.2286 (2)	0.04393 (6)	0.0290 (2)	
H6	0.2500 (9)	−0.359 (2)	0.0595 (8)	0.031 (3)*	
C7	0.07032 (8)	0.0524 (3)	−0.13467 (7)	0.0394 (3)	
H7A	0.0142 (12)	0.144 (3)	−0.1426 (10)	0.051 (5)*	
H7B	0.0508 (15)	−0.099 (4)	−0.1622 (13)	0.086 (7)*	
C8	0.12580 (9)	0.1689 (3)	−0.17069 (7)	0.0451 (3)	
H8	0.1781 (14)	0.088 (3)	−0.1728 (11)	0.066 (5)*	
C9	0.10758 (12)	0.3641 (4)	−0.20373 (8)	0.0597 (5)	
H9	0.0469 (18)	0.449 (4)	−0.2068 (14)	0.099 (8)*	
H9B	0.1442 (14)	0.434 (4)	−0.2293 (12)	0.071 (6)*	
C10	0.28746 (7)	−0.31981 (18)	0.19593 (6)	0.0249 (2)	
H10A	0.2564 (9)	−0.465 (2)	0.1772 (8)	0.028 (3)*	
H10B	0.3377 (9)	−0.304 (2)	0.1801 (7)	0.026 (3)*	
C11	0.31793 (6)	−0.31470 (17)	0.27769 (6)	0.0230 (2)	
C12	0.32669 (7)	−0.13692 (17)	0.32445 (6)	0.0232 (2)	
H12	0.3179 (9)	0.025 (2)	0.3173 (8)	0.031 (3)*	
C13	0.38036 (6)	−0.12328 (16)	0.46256 (5)	0.0210 (2)	
C14	0.44278 (7)	−0.22392 (18)	0.52340 (6)	0.0246 (2)	
H14	0.4712 (9)	−0.363 (2)	0.5174 (8)	0.029 (3)*	
C15	0.46548 (7)	−0.12254 (18)	0.59111 (6)	0.0249 (2)	
H15	0.5101 (11)	−0.194 (3)	0.6325 (9)	0.040 (4)*	
C16	0.42651 (6)	0.08019 (17)	0.59909 (5)	0.0226 (2)	
C17	0.36412 (7)	0.17993 (17)	0.53822 (6)	0.0240 (2)	
H17	0.3362 (10)	0.328 (3)	0.5429 (8)	0.039 (4)*	
C18	0.34120 (7)	0.07693 (17)	0.46974 (5)	0.0232 (2)	
H18	0.2975 (8)	0.148 (2)	0.4265 (7)	0.025 (3)*	
C19	0.41329 (10)	0.3629 (2)	0.68101 (7)	0.0373 (3)	
H19A	0.3554 (13)	0.341 (3)	0.6731 (10)	0.050 (5)*	
H19B	0.4447 (11)	0.399 (3)	0.7326 (10)	0.047 (4)*	
H19C	0.4223 (12)	0.480 (3)	0.6507 (10)	0.056 (5)*	
C20	0.07124 (9)	0.3989 (2)	0.10918 (8)	0.0374 (3)	
H20A	0.0124 (13)	0.347 (3)	0.0825 (11)	0.060 (5)*	
H20B	0.0740 (10)	0.471 (3)	0.1585 (10)	0.047 (4)*	
H20C	0.0901 (10)	0.514 (3)	0.0820 (9)	0.046 (4)*	

### Atomic displacement parameters (Å^2^)

**Table:**

	*U*^11^	*U*^22^	*U*^33^	*U*^12^	*U*^13^	*U*^23^
O1	0.0318 (4)	0.0271 (4)	0.0177 (4)	0.0090 (3)	0.0046 (3)	0.0019 (3)
O2	0.0408 (5)	0.0298 (4)	0.0294 (4)	0.0122 (3)	0.0088 (3)	0.0000 (3)
O3	0.0365 (4)	0.0303 (4)	0.0194 (4)	0.0041 (3)	0.0036 (3)	−0.0023 (3)
N1	0.0260 (4)	0.0191 (4)	0.0178 (4)	0.0022 (3)	0.0048 (3)	0.0024 (3)
N2	0.0424 (5)	0.0192 (4)	0.0227 (4)	0.0025 (4)	0.0063 (4)	0.0018 (3)
N3	0.0394 (5)	0.0216 (4)	0.0222 (4)	0.0017 (4)	0.0051 (4)	0.0006 (3)
C1	0.0232 (4)	0.0261 (5)	0.0187 (5)	0.0007 (4)	0.0035 (3)	0.0012 (4)
C2	0.0241 (5)	0.0244 (5)	0.0251 (5)	0.0012 (4)	0.0057 (4)	0.0009 (4)
C3	0.0269 (5)	0.0297 (5)	0.0272 (5)	0.0027 (4)	0.0025 (4)	0.0060 (4)
C4	0.0275 (5)	0.0396 (6)	0.0217 (5)	−0.0023 (4)	0.0018 (4)	0.0041 (4)
C5	0.0338 (6)	0.0409 (6)	0.0209 (5)	0.0036 (5)	0.0051 (4)	−0.0023 (5)
C6	0.0289 (5)	0.0317 (5)	0.0229 (5)	0.0057 (4)	0.0045 (4)	−0.0008 (4)
C7	0.0349 (6)	0.0502 (8)	0.0241 (6)	−0.0033 (5)	−0.0010 (4)	0.0065 (5)
C8	0.0337 (6)	0.0741 (10)	0.0234 (6)	0.0000 (6)	0.0047 (5)	0.0015 (6)
C9	0.0578 (9)	0.0807 (12)	0.0296 (7)	−0.0256 (9)	0.0014 (6)	0.0125 (7)
C10	0.0273 (5)	0.0240 (5)	0.0199 (5)	0.0057 (4)	0.0040 (4)	0.0003 (4)
C11	0.0238 (4)	0.0222 (5)	0.0207 (5)	0.0020 (3)	0.0051 (4)	0.0015 (4)
C12	0.0267 (5)	0.0221 (5)	0.0189 (5)	0.0035 (4)	0.0055 (4)	0.0036 (4)
C13	0.0240 (4)	0.0206 (4)	0.0170 (4)	−0.0005 (3)	0.0055 (3)	0.0017 (3)
C14	0.0270 (5)	0.0238 (5)	0.0217 (5)	0.0059 (4)	0.0070 (4)	0.0035 (4)
C15	0.0258 (5)	0.0264 (5)	0.0196 (5)	0.0043 (4)	0.0044 (4)	0.0042 (4)
C16	0.0247 (4)	0.0234 (5)	0.0185 (4)	−0.0017 (3)	0.0061 (3)	0.0005 (4)
C17	0.0287 (5)	0.0194 (4)	0.0225 (5)	0.0020 (4)	0.0074 (4)	0.0016 (4)
C18	0.0263 (5)	0.0206 (4)	0.0197 (5)	0.0024 (3)	0.0045 (4)	0.0042 (4)
C19	0.0553 (8)	0.0269 (6)	0.0247 (6)	0.0042 (5)	0.0079 (5)	−0.0040 (4)
C20	0.0363 (6)	0.0290 (6)	0.0471 (7)	0.0098 (5)	0.0148 (5)	0.0040 (5)

### Geometric parameters (Å, º)

**Table:**

O1—C1	1.3712 (12)	C8—H8	0.99 (2)
O1—C10	1.4279 (12)	C9—H9	1.09 (3)
O2—C2	1.3673 (13)	C9—H9B	0.99 (2)
O2—C20	1.4220 (14)	C10—C11	1.4908 (14)
O3—C16	1.3631 (12)	C10—H10A	1.002 (14)
O3—C19	1.4213 (15)	C10—H10B	0.971 (14)
N1—N2	1.3504 (13)	C11—C12	1.3708 (15)
N1—C12	1.3541 (13)	C12—H12	0.978 (15)
N1—C13	1.4315 (13)	C13—C18	1.3812 (14)
N2—N3	1.3142 (13)	C13—C14	1.3942 (14)
N3—C11	1.3600 (13)	C14—C15	1.3764 (15)
C1—C6	1.3814 (15)	C14—H14	0.975 (14)
C1—C2	1.4082 (14)	C15—C16	1.3969 (15)
C2—C3	1.3827 (15)	C15—H15	0.970 (17)
C3—C4	1.3991 (17)	C16—C17	1.3921 (14)
C3—H3	0.974 (16)	C17—C18	1.3935 (14)
C4—C5	1.3808 (17)	C17—H17	1.010 (16)
C4—C7	1.5185 (16)	C18—H18	0.984 (13)
C5—C6	1.3995 (16)	C19—H19A	0.905 (19)
C5—H5	0.972 (17)	C19—H19B	0.977 (18)
C6—H6	0.964 (15)	C19—H19C	0.956 (19)
C7—C8	1.491 (2)	C20—H20A	0.96 (2)
C7—H7A	1.025 (18)	C20—H20B	1.037 (18)
C7—H7B	1.04 (2)	C20—H20C	0.979 (17)
C8—C9	1.312 (2)		
			
O1···O2	2.5723 (13)	C18···H12	2.871 (15)
O1···H12	2.870 (14)	C19···H17	2.543 (15)
O2···H10A^i^	2.681 (14)	C19···O1^vi^	3.3285 (19)
O3···H19B^ii^	2.579 (18)	C20···H3	2.508 (15)
N2···C18^iii^	3.3320 (15)	C20···H10A^i^	2.938 (15)
N2···H14	2.532 (15)	H3···H7A	2.43 (2)
N2···H18^iii^	2.864 (13)	H3···H20A	2.38 (3)
N2···H14^iv^	2.812 (15)	H3···H20C	2.31 (2)
N3···H12^iii^	2.834 (12)	H5···H7B	2.52 (3)
N3···H15^iv^	2.848 (18)	H6···H10A	2.34 (2)
C12···C15^v^	3.5436 (18)	H6···H10B	2.30 (2)
C13···C15^v^	3.3633 (17)	H6···C17^ix^	2.791 (14)
C13···C14^v^	3.4679 (17)	H6···C18^ix^	2.955 (15)
C14···C14^v^	3.5463 (18)	H7A···H9	2.37 (3)
C14···C18^v^	3.5668 (18)	H7B···H9^x^	2.50 (4)
C19···C1^vi^	3.589 (2)	H8···N3^ix^	2.808 (1)
C19···C10^vi^	3.4746 (19)	H9···H20B^xi^	2.50 (3)
C1···H20C^iii^	2.856 (18)	H9B···O2^xii^	2.837 (5)
C3···H20C	2.792 (17)	H10B···C16^ix^	2.976 (14)
C3···H20A	2.82 (2)	H10B···C19^xii^	2.897 (13)
C4···H20A^vii^	2.88 (2)	H12···H18	2.377 (19)
C5···H20A^vii^	2.98 (2)	H14···H14^iv^	2.107 (19)
C6···H20C^iii^	2.811 (17)	H15···H19C^iii^	2.51 (3)
C6···H10A	2.814 (4)	H15···H19B^ii^	2.53 (2)
C6···H10B	2.748 (13)	H17···H19A	2.44 (2)
C9···H7B^viii^	2.96 (2)	H17···H19C	2.26 (2)
C10···H6	2.517 (15)	H18···C8^vi^	2.970 (13)
C12···H18	2.776 (13)	H19A···O1^vi^	2.67 (2)
C15···H19C^iii^	2.830 (18)	H19A···C1^vi^	2.91 (2)
C17···H19C	2.725 (18)	H19C···H10B^vi^	2.55 (2)
C17···H19A	2.843 (19)		
			
C1—O1—C10	117.19 (8)	C11—C10—H10A	110.0 (8)
C2—O2—C20	117.19 (10)	O1—C10—H10B	109.5 (8)
C16—O3—C19	117.42 (9)	C11—C10—H10B	109.6 (8)
N2—N1—C12	110.77 (8)	H10A—C10—H10B	109.9 (11)
N2—N1—C13	119.89 (8)	N3—C11—C12	108.73 (9)
C12—N1—C13	129.33 (9)	N3—C11—C10	121.31 (9)
N3—N2—N1	107.21 (8)	C12—C11—C10	129.94 (9)
N2—N3—C11	108.86 (9)	N1—C12—C11	104.42 (9)
O1—C1—C6	125.33 (9)	N1—C12—H12	121.7 (8)
O1—C1—C2	115.27 (9)	C11—C12—H12	133.8 (8)
C6—C1—C2	119.40 (10)	C18—C13—C14	120.55 (9)
O2—C2—C3	125.30 (10)	C18—C13—N1	120.54 (8)
O2—C2—C1	115.02 (9)	C14—C13—N1	118.91 (9)
C3—C2—C1	119.68 (10)	C15—C14—C13	119.62 (10)
C2—C3—C4	121.15 (10)	C15—C14—H14	120.6 (8)
C2—C3—H3	118.9 (9)	C13—C14—H14	119.7 (8)
C4—C3—H3	119.9 (9)	C14—C15—C16	120.42 (9)
C5—C4—C3	118.60 (10)	C14—C15—H15	118.3 (9)
C5—C4—C7	121.24 (11)	C16—C15—H15	121.3 (9)
C3—C4—C7	120.15 (11)	O3—C16—C17	125.29 (9)
C4—C5—C6	120.98 (11)	O3—C16—C15	114.93 (9)
C4—C5—H5	119.5 (10)	C17—C16—C15	119.78 (9)
C6—C5—H5	119.5 (10)	C16—C17—C18	119.70 (9)
C1—C6—C5	120.15 (10)	C16—C17—H17	120.7 (9)
C1—C6—H6	119.3 (9)	C18—C17—H17	119.6 (9)
C5—C6—H6	120.5 (9)	C13—C18—C17	119.94 (9)
C8—C7—C4	114.32 (10)	C13—C18—H18	120.2 (8)
C8—C7—H7A	109.4 (10)	C17—C18—H18	119.8 (8)
C4—C7—H7A	110.7 (10)	O3—C19—H19A	111.9 (11)
C8—C7—H7B	106.6 (13)	O3—C19—H19B	104.5 (10)
C4—C7—H7B	108.6 (13)	H19A—C19—H19B	110.0 (15)
H7A—C7—H7B	106.9 (16)	O3—C19—H19C	108.7 (11)
C9—C8—C7	125.01 (16)	H19A—C19—H19C	111.8 (15)
C9—C8—H8	117.5 (12)	H19B—C19—H19C	109.6 (15)
C7—C8—H8	117.4 (12)	O2—C20—H20A	111.5 (11)
C8—C9—H9	118.7 (14)	O2—C20—H20B	105.0 (9)
C8—C9—H9B	123.1 (13)	H20A—C20—H20B	110.1 (14)
H9—C9—H9B	118.0 (18)	O2—C20—H20C	111.3 (9)
O1—C10—C11	106.67 (8)	H20A—C20—H20C	111.8 (15)
O1—C10—H10A	111.1 (8)	H20B—C20—H20C	106.8 (14)
			
C12—N1—N2—N3	−0.64 (12)	N2—N3—C11—C12	−0.22 (12)
C13—N1—N2—N3	179.75 (8)	N2—N3—C11—C10	178.55 (9)
N1—N2—N3—C11	0.52 (12)	O1—C10—C11—N3	154.23 (9)
C10—O1—C1—C6	2.77 (15)	O1—C10—C11—C12	−27.29 (15)
C10—O1—C1—C2	−178.10 (9)	N2—N1—C12—C11	0.49 (11)
C20—O2—C2—C3	5.04 (16)	C13—N1—C12—C11	−179.94 (9)
C20—O2—C2—C1	−174.39 (10)	N3—C11—C12—N1	−0.16 (11)
O1—C1—C2—O2	2.06 (14)	C10—C11—C12—N1	−178.80 (10)
C6—C1—C2—O2	−178.75 (10)	N2—N1—C13—C18	−155.77 (10)
O1—C1—C2—C3	−177.40 (9)	C12—N1—C13—C18	24.69 (15)
C6—C1—C2—C3	1.79 (16)	N2—N1—C13—C14	25.16 (14)
O2—C2—C3—C4	−179.27 (10)	C12—N1—C13—C14	−154.37 (10)
C1—C2—C3—C4	0.13 (16)	C18—C13—C14—C15	−0.03 (15)
C2—C3—C4—C5	−1.79 (17)	N1—C13—C14—C15	179.04 (9)
C2—C3—C4—C7	177.07 (10)	C13—C14—C15—C16	−0.10 (16)
C3—C4—C5—C6	1.55 (18)	C19—O3—C16—C17	3.73 (16)
C7—C4—C5—C6	−177.30 (11)	C19—O3—C16—C15	−176.25 (10)
O1—C1—C6—C5	177.07 (10)	C14—C15—C16—O3	−179.83 (9)
C2—C1—C6—C5	−2.03 (17)	C14—C15—C16—C17	0.19 (15)
C4—C5—C6—C1	0.35 (18)	O3—C16—C17—C18	179.87 (9)
C5—C4—C7—C8	−80.52 (17)	C15—C16—C17—C18	−0.16 (15)
C3—C4—C7—C8	100.66 (15)	C14—C13—C18—C17	0.06 (15)
C4—C7—C8—C9	−121.28 (16)	N1—C13—C18—C17	−178.99 (9)
C1—O1—C10—C11	176.23 (8)	C16—C17—C18—C13	0.03 (15)

Symmetry codes: (i) *x*, *y*+1, *z*; (ii) −*x*+1, *y*−1/2, −*z*+3/2; (iii) *x*, *y*−1, *z*; (iv) −*x*+1, −*y*−1, −*z*+1; (v) −*x*+1, −*y*, −*z*+1; (vi) *x*, −*y*+1/2, *z*+1/2; (vii) −*x*, −*y*, −*z*; (viii) −*x*, *y*+1/2, −*z*−1/2; (ix) *x*, −*y*−1/2, *z*−1/2; (x) −*x*, *y*−1/2, −*z*−1/2; (xi) −*x*, −*y*+1, −*z*; (xii) *x*, −*y*+1/2, *z*−1/2.

### Hydrogen-bond geometry (Å, º)

*Cg*3 is the centroid of the benzene ring *C* (C13–C18).

**Table:**

*D*—H···*A*	*D*—H	H···*A*	*D*···*A*	*D*—H···*A*
C6—H6···*Cg*3^xiii^	0.964 (15)	2.825 (15)	3.5168 (15)	129.4 (11)
C19—H19*B*···O3^xiv^	0.977 (18)	2.578 (18)	3.4587 (16)	150.0 (14)

Symmetry codes: (xiii) *x*, −*y*−3/2, *z*−3/2; (xiv) −*x*+1, *y*+1/2, −*z*+3/2.
